# Borderless Teleradiology with CHILI

**DOI:** 10.2196/jmir.1.2.e8

**Published:** 1999-12-13

**Authors:** Uwe Engelmann, A Schroeter, M Schwab, U Eisenmann, M Vetter, K Lorenz, J Quiles, I Wolf, H Evers, HP Meinzer

**Affiliations:** ^1^Deutsches KrebsforschungszentrumAbteilung Mediziniscdhe und Biologische InformatikImNeuenheimer Feld 280D-69120HeidelbergGermany; ^2^Steinbeis-Transferzentrum Medizinische InformatikHeidelbergGermany; ^3^University of Santiago de CompostelaDepartment of RadiologySpain

**Keywords:** Teleradiology, Telemedicine, Remote Consultation, Diagnostic Imaging, Computer-Assisted Image Interpretation, PACS, Middleware, TLS, Security, Plugin, Visualization

## Abstract

Teleradiology is one of the most evolved areas of telemedicine, but one of the basic problems which remains unsolved concerns system compatibility. The DICOM (Digital Imaging and Communications in Medicine) standard is a prerequisite, but it is not sufficient in all aspects. Examples of other currently open issues are security and cooperative work in synchronous teleconferences. Users without a DICOM radiological workstation would benefit from the ability to join a teleradiology network without any special tools. Drawbacks of many teleradiology systems are that they are monolithic in their software design and cannot be adapted to the actual user's environment. Existing radiological systems currently cannot be extended with additional software components. Consequently, every new application usually needs a new workstation with a different look and feel, which must be connected and integrated into the existing infrastructure.

This paper introduces the second generation teleradiology system CHILI. The system has been designed to match both the teleradiology requirements of the American College of Radiology (ACR), and the functionality and usability needs of the users. The experiences of software developers and teleradiology users who participated in the first years of the clinical use of CHILI's predecessor MEDICUS have been integrated into a new design. The system has been designed as a component-based architecture. The most powerful communication protocol for data exchange and teleconferencing is the CHILI protocol, which includes a strong data security concept. The system offers, in addition to its own secure protocol, several different communication methods: DICOM, classic e-mail, Remote Copy functions (RCP), File Transfer Protocol (FTP), the internet protocols HTTP (HyperText Transfer Protocol) and HTTPS (HyperText Transfer Protocol Secure),and CD-ROMs for off-line communication. These transfer methods allow the user to send images to nearly anyone with a computer and a network. The drawbacks of the non-CHILI protocols are that teleconferences are not possible, and that the user must take reasonable precautions for data privacy and security.

The CHILI PlugIn mechanism enables the users or third parties to extend the system capabilities by adding powerful image postprocessing functions or interfaces to other information systems. Suitable PlugIns can be either existing programs, or dedicated applications programmed with interfaces to the CHILI components. The developer may freely choose programming languages and interface toolkits.

The CHILI architecture is a powerful and flexible environment for Picture Archiving and Communications Systems (PACS)and teleradiology. More than 40 systems are currently running in clinical routine in Germany. More than 300,000 images have been distributed among the communication partners in the last two years. Feedback and suggestions from the users influenced the system architecture by a great extent. The proposed and implemented system has been optimized to be as platform independent, open, and secure as possible.

## Introduction

The American College of Radiology (ACR) defined teleradiology in *ACR Standard for Teleradiology* [1] as the following:

Teleradiology is the electronic transmission of radiological images from one location to another for the purposes of interpretation and/or consultation. Teleradiology may allow even more timely interpretation of radiological images and give greater access to secondary consultations and to improved continuing education. Users in different locations may simultaneously view images. Appropriately utilized, teleradiology can improve access to quality radiological interpretations and thus significantly improve patient care.

In this paper we want to introduce the current state of our development in teleradiology. CHILI (tm) is a general purpose radiology workstation with additional functions for teleradiology. The task of the system is the transmission of radiological images to different locations for interpretation and consultation. The transmitted images can be simultaneously viewed at different locations and examined cooperatively. The development of the system was based on experience with the successful teleradiology project MEDICUS-2, which was a dedicated teleradiology system [2]. The second generation teleradiology system CHILI has been designed to match the teleradiology requirements of the ACR and the needs of MEDICUS users [3]. The experiences of software developers and teleradiology users who participated in the first years of clinical use have been integrated into the architecture [4,5]. This paper describes the general system design and application areas.

## Methods

### Principal Functionality

The principal functions of the system are the following:

- The system receives images from different sources, such as imaging modalities, radiological viewing stations, digital archives, video cameras, etc., and stores them in a local database management system.
- The image viewing functionality allows the user to display and analyze the images in several ways (e.g. level/windowing, measurements, ROI analysis).
- Images can be transmitted to other sites with a number of (vendor-independent) protocols. Security measures are taken into account. The network protocol is TCP/IP and independent of the physical network layer. Different protocols are available.
- Images can be compressed using wavelet-based algorithms.
- Users at remote locations can work on the images simultaneously in teleconferences with synchronized images, functions, and mouse pointers.
- Exporting images to hard disk, floppy, or CD-ROM is possible, and hardcopies of the images can be printed on inexpensive, ordinary PostScript printers. Also, the DICOM protocol can be used to perform printing management in combination with DICOM compliant print servers.

### Component Architecture

The software architecture of CHILI is based on the results of the European Project HELIOS, in which concepts for distributed, object-oriented software systems were developed [6]. The core of the HELIOS software engineering environment is the HELIOS Unification Bus (HUB), a middleware technology. Independent and distributed software components communicate by means of the HUB. The middleware of CHILI is based on the same concepts as HELIOS, but has been redesigned in several aspects to match the specific requirements of an image communication environment that has parts which are linked by low bandwidth networks and are not permanently reachable. Security enhancements have been added to fulfill the legal requirements for data security and privacy. The middleware supports the security standards SSL/TLS (Secure Sockets Layer/Transport Layer Security) for authentication and secure communication [7].

**Figure 1:**
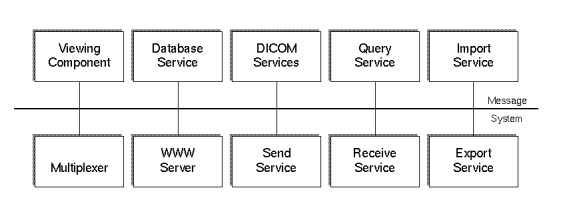
shows the main components of the architecture

Figure 1 shows the main components of the architecture:

- The *Viewer* is the graphical interface for the user. Nearly all functions are controlled by the viewer, including viewing images, transmission of images, teleconferences, and system configuration.
- The *Message System* is used as the communication channel (middleware) between the software components.
- The *Database Service* component stores the data for the system and delivers it to other software components. It provides an abstract interface which hides the specific SQL database management system (DBMS). Thus, it is independent of a specific DBMS implementation. DICOM (Digital Imaging and Communications in Medicine) archives can be accessed through this interface as well. This is a great advantage both for users and programmers.
- *Send and Receive Services* are used to exchange data between the CHILI systems via the CHILI protocol. Other protocols are available as well and allow data exchange with non-CHILI systems (see Cross-Platform Communication). An optional wavelet module (MT-Wice, MeVis Technology, Bremen, Germany) can be employed to compress the image by a factor of 10-100 to speed up the transmission process.
- The *Import and Export Services* are needed to get the data into the system and to export data to the local disk or an archive medium. DICOM is the most relevant image format for this purpose. The older ACR/NEMA 1.0 and 2.0 standards; industry standards; and public domain formats such as GIF, TIFF, PostScript, PPM, JPEG, M-JPEG are supported as well. Vendor specific formats, like SOMATOM, MAGNETOM, and GENESIS, can also be processed.
- The *DICOM Server* receives image data via the DICOM protocol as a Storage Class Provider from other DICOM devices, such as CT or MRI scanners.
- The *Query Service* is based on the DICOM protocol to retrieve image data from Picture Archiving and Communications Systems (PACS) archive systems.
- The *Multiplexer* manages connection requests between the different components and starts modules when necessary.
- *The World Wide Web (WWW) Server* is the gateway between the CHILI database and internet technology (see WWW: Independent of Hardware and Operating System).

This overall design allows a very flexible configuration of the system which can easily be adapted to any specific environment. The different services and modules can be combined in different ways to provide custom solutions. Examples of typical configurations (in clinical use) are:

- *CHILI Classic*, with full functionality ranging from DICOM image database to printing and teleconferencing.
- *CHILI Video* is a package without connections to modalities or databases but with on-line communications using digital images which are grabbed from a video source.
- *Send and Receive systems* can be used to perform one-way communications setups.
- *Client/Server* configurations use a central PACS archive or CHILI database and allow the transmission of data to remote sites as well as teleconferences. The server can either be a CHILI Classic (with or without viewer) or a database server (SQL and/or DICOM). The clients can either be viewers or complete systems which share a central database. All clients have the complete functionality of a CHILI Classic configuration in connection with a server. Other dedicated systems can be realized depending on the actual user needs.

### Security Concept

The most powerful communication protocol for data exchange (and the only protocol that allows teleconferencing) is the CHILI protocol, which includes a strong data security concept [8]. This includes all measures which are necessary to comply with German and European requirements and laws. As a result of the data security concept, it is possible to restrict the rights of the receiver on the image data. The following restrictions are possible:

1. Only viewable in a teleconference with the sender.
2. Not exportable.
3. Not printable.
4. Automatic removal after the teleconference.

All data packets to be transmitted are encrypted with the PGP (Pretty Good Privacy) public key encryption system [9]. The public key of the receiver is used for the encryption. A checksum of the data is calculated and signed with the digital signature of the sender. This protects data integrity, and allows for authentication of the sender and privacy. Furthermore, all local data are encrypted with a symmetric key encryption method. Transmitted and received images are logged in special log files which are protected with a checksum.

The communication between the CHILI components via the middleware can optionally be protected using the TLS standard [7]. This ensures authentication and privacy.

### Cross-Platform Communication

**Figure 2:**
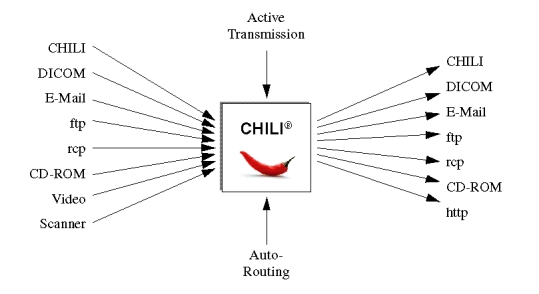
CHILI offers additional communication methods

It cannot be expected that all communication partners have the same teleradiology system. Thus, CHILI offers additional communication methods (Figure 2):

- The *DICOM protocol* allows the exchange of images with all other DICOM compliant systems. The following service classes are supported: storage class user, storage class provider, and print management classes for both user and provider. Find and Move are also supported.
- Classic *e-mail*(SMTP protocol) can be used to send images encoded in either DICOM or JPEG formats.
- *File Transfer Protocol*(FTP) and *Remote Copy functions*(RCP) are available to transmit image data to other hosts where the data is processed with other software tools.
- Data can be transferred to *portable media*, such as CD-ROMs (DICOMDIR). The CD-ROMS are suitable for archiving purposes as well. The database stores a reference to the CD-ROM where the data are actually stored. The media can be handled manually or automatically with a jukebox. Non-archive CD-ROMs can be given to other medical institutions, referring physicians, or even the patient. The standardized DICOMDIR format ensures that the data can be viewed with different programs which are compliant to the standard.
- Images or image sequences can be acquired from *Video* sources, such as video camera, video recorder, and ultrasound devices. The system converts the analog video signal into DICOM compliant image files. Demographic data is acquired as well to facilitate data-handling in the patient-oriented database and long-term PACS archives.
- Data from document or film scanners can be imported in different formats and converted to the DICOM standard for further processing.
- The WWW server (see Component Architecture) translates the internal SQL or DICOM representation of the data to the HTTP protocol. Thus, the data can be accessed with any web browser in the network. The advantage of this solution is that the clients do not need any special hardware or software. Authenticity of users and privacy are ensured by different measures, such as user accounts, passwords, restricted group access, and the secure protocol TLS.

The user of the CHILI system is not confronted with the various protocols directly. The definition of the protocol to be used is configured for each communication partner. It has to be defined if the partner is connected via LAN, Internet, or ISDN lines. The ISDN zone is defined as well to be able to estimate the transfer costs depending of the transmission time, duration, distance, and compression rates.

The broad set of transfer methods enables the CHILI user to send images to nearly anyone with access to a computer and a network. The limitations are that teleconferences are possible with the CHILI protocol only, and that the user has to take reasonable precautions for data privacy and security when he does not use the secure CHILI protocol.

### Explicit Transmissionand Auto-routing

Image data can be transmitted manually by the user or automatically by the auto-routing component which is activated by the import service. Certain data fields of the image header are used to determine if and to whom the incoming data should be transmitted automatically.

### Functional Openness

A typical problem in radiology is that nearly every application needs its own computer. Console computers are necessary to operate the imaging modalities. Viewing stations are used for reporting or further analysis of the image data. Radiology Information System (RIS) terminals are used to manage and organize the patient information flow. Additional Personal Computers (PCs) are used for word processing (e.g. reports, publications). New computers for teleradiology are now entering the radiology department.

Developers of new medical image analysis applications face the problem that the current computer applications (e.g. viewing stations) are not open enough. Every developer must consider the connection to the modality or viewing station, file formats, data storage of files on the disk or in a data base system, and the display of 12-bit images on 8-bit screens. For the radiologist, the development a new method means the invention of many wheels a second or third time before the real problems can be addressed. Most of the manufacturers of the existing viewing stations do not allow the installation of additional software. Usually the customers do not get the root password of the systems. An additional computer with different user interfaces and additional interfaces to the rest of the world has to be introduced, therefore, to get the new functionality to the users.

**Figure 3:**
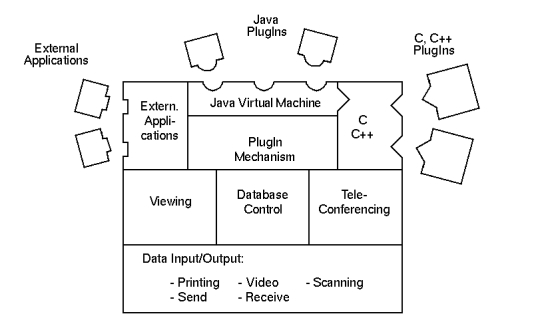
PlugIns can be either existing applications or new modules with interfaces to the existing CHILI components

The result of the depicted problem is that it takes a lot of effort for developers to implement and introduce new methods; this is most of the time very costly for the end user. Therefore, we designed the CHILI PlugIn mechanism [10]. Users can extend the system by adding powerful image postprocessing functions [11] or interfaces to other information systems. PlugIns can be either existing applications or new modules with interfaces to the existing CHILI components (Figure 3).

The PlugIn developers' Application Programming Interface (API) includes interfaces to all software components and the message system [10]. The developer is free to choose programming languages and interface toolkits (e.g. C, C++, Tcl/Tk). The Java Virtual Machine [12] is a PlugIn itself; thus, Java programs can easily be integrated to communicate with CHILI.

**Figure 4:**
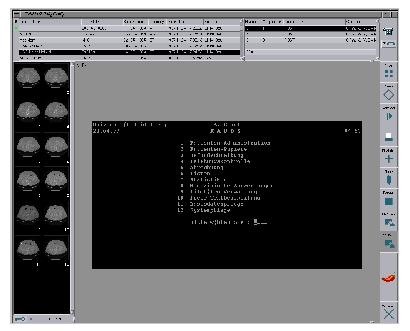
This enables the user to see both the image data and RIS information on the same screen

Several existing applications have successfully been integrated into CHILI as useful additions to the main system. One example is a terminal window (xterm) which is connected to a RADOS-M RIS. This enables the user to see both the image data and RIS information on the same screen (Figure 4). Another example of such a simple PlugIn is a WWW browser, which allows the user to explore, for example, reference cases on the intra- or internet. The user manual of the system is integrated with the same mechanism.

**Figure 5:**
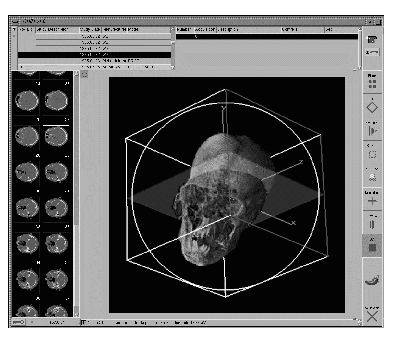
Examples for ongoing PlugIn development projects are a general purpose 3D volume segmentation and visualization project

Examples for ongoing PlugIn development projects are a general purpose 3D volume segmentation and visualization project [13] (Figure 5), a tool for the planning of liver surgeries [14], and a 3D ultrasound doppler project [15].

### Hardware and Operating Systems

#### Unix-Workstations and Personal Computers with Linux

CHILI has been developed under the UNIX operating system in ANSI C. The most important reasons were portability, reliability, and data security. The realized system is not dependent on vendor-specific UNIX dialects or hardware features. It is available on workstations running IRIX, Solaris, Ultrix, and Linux. The most economically priced and powerful system is a PC running the Linux operating system [16].

#### Personal Computers with Microsoft Windows

CHILI clients are running under Microsoft Windows under the eXceed system (Hummingbird). The latter allows the original code to run and provides a X Window-based user interface. It offers the same functionality as the Unix client, including teleconferences.

A complete CHILI Classic configuration has been realized using Interix (formerly known as OpenNT, by Softway Systems, Inc.) on Windows NT systems, with all components for data exchange and teleconferencing operational.

#### WWW: Independent of Hardware and Operating System

The Web Interface is the most hardware and operating system independent solution. The CHILI WWW Server is a frontend to the CHILI database. Users can access it with any web browser. The user interface is nearly the same as the standard CHILI Viewer. Data can be retrieved from CHILI databases, or DICOM archives which are accessed through the CHILI database interface. The images can be displayed with 8-bit screens in GIF or JPEG format, and DICOM encoded for the Java/DICOM viewer. The compression rate of the JPEG images can be defined in different quality steps.

### From Teleradiology to PACS

**Figure 6:**
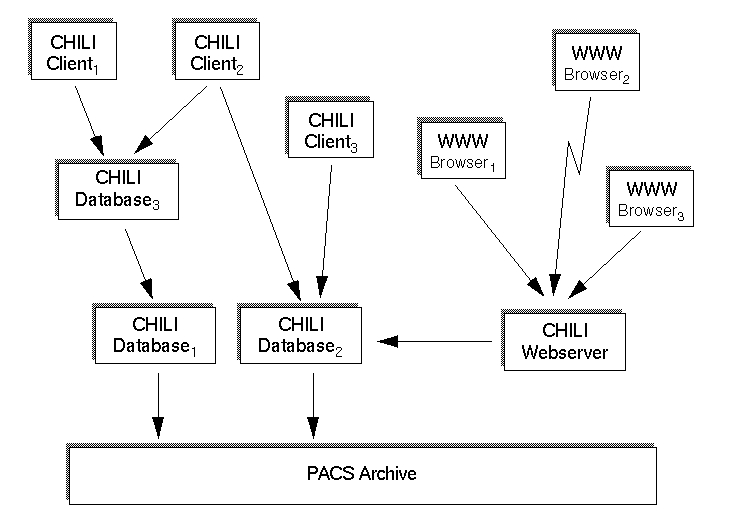
The CHILI database can be configured to act as a cache for the PACS archive

Over time, the system has become a PACS component which can be used as an interface to DICOM compliant archives, and also for DICOM printing and reporting. Images can be distributed in-house, and light-weight clients running on PCs can be used to view or process the images in teleconferences. Clients for MS Windows are available, in addition to web-based interfaces which can be accessed from any hardware or operating system with a web browser. The CHILI database can be configured to act as a cache for the PACS archive (Figure 6). A storage hierarchy can be configured to reduce the traffic on the archive and to optimize image transfers on the network. The user is released from the load of knowing where the data are actually stored.

The CHILI Viewer with the multi-head option and diagnostic monitors is suited for image reporting. Filmless radiology can be realized with this technology.

## Results

The CHILI project is a cooperation between the German Cancer Research Center and a technology transfer company Steinbeis-Transferzentrum Medizinische Informatik (STZ-MI), both located in Heidelberg, Germany. The STZ-MI is an outgrowth of the cancer center where scientific results are converted into products for market. Concepts and research are done by the research group at the cancer center, and the technology transfer company implements the research into products and brings the results to the market. Marketing and sales is also done by OEM partners, system integrators, and consulting firms.

More than 40 systems are running in daily routine in private practices, small hospitals, university clinics, and research institutes. The system is in use in research projects, in medical image analysis, and even surgery planning systems. It is the basic platform of the German research project Sonderforschungsbereich 414 *Information Technology in Medicine: Computer and Sensor Supported Surgery* [17].

**Figure 7:**
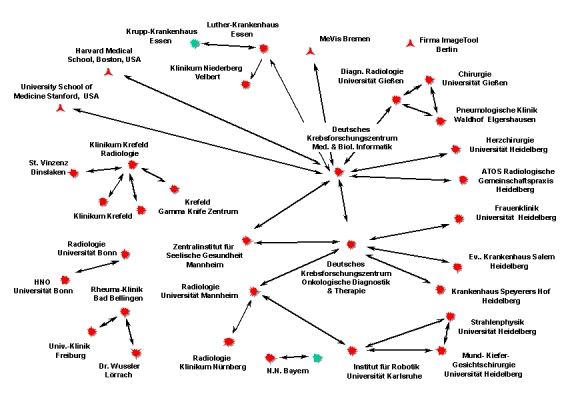
shows the current network (July 1999)

Figure 7 shows the current network (July 1999). More than 300,000 images have been processed by the network, and the program has been invoked nearly 20,000 times in the last 3 years. More than twenty modalities from different vendors are connected via DICOM or proprietary solutions [18].

The system is in use in different application scenarios, such as image delivery to referring physicians, interdisciplinary discussions, remote reporting, de-centralized radiology departments, resource sharing, data transfer for radiotherapy planning, scientific cooperation, quality control, and research.

## Discussion

### Comparison with Work of Others

An overview about the state of teleradiology in the United States has been published by Grigsby in 1994 [19]. Regular telephone lines and modems were normally used at that time in the US. Most of the systems developed were based on PCs using video cameras with PC frame grabber cards or scanners to capture the images, e.g [19,20].. Oft-discussed topics in the field of teleradiology were spatial resolution and grayvalue depth of the video images or scanners. However, these problems do not exist when the original digital images are processed; the receiver gets the images in original quality. Such advanced teleradiology systems based on digital data and digital telephone lines (ISDN) have been developed and used in Europe since 1994, e.g [20-24]..

A prototype of a dedicated teleradiology system has been developed by Gomez and co-workers in a research project funded by the EU AIM Programme [21]. The system has been evaluated in two hospitals in Spain. One imaging modality was connected to one of two teleradiology workstations. Handels describes another German teleradiology system, called KAMEDIN, which has been used in a field test for several months but has never gone to clinical routine [22].

All major image modality vendors in the field of radiology have been talking about teleradiology since 1994 or 1995. The main difference of the industrial solutions is that they simply extend the local area network of the radiology department by an ISDN link to another location. Images are then sent with proprietary protocols (sometimes with DICOM) to a remote machine. Teleconferences with two users at different locations who work together on the same images are not possible. Security aspects are missing as well.

CHILI (and its predecessor MEDICUS) seems to be the only system which has a complete data security concept [8]. A great advantage of the system, compared to others, is the direct connection with the imaging modalities of different vendors. The DICOM protocol can be used to receive the images, or proprietary solutions can be enabled for older modalities without DICOM interfaces.

A major feature of CHILI is that it is not a dedicated teleradiology system, such as its prototype MEDICUS or the KAMEDIN system [22]. Instead, it is a general purpose multimodality workstation with strong teleradiology and security features. Cross-platform communication, vendor independence, and extensibility are major distinctive features in comparisons. The system is also used for advanced image analysis functions and image reporting.

New teleradiology systems are currently under development in the programming language Java [12]. This makes the systems independent of the operating system and hardware used. Examples are the CYPRIS system which is under development by Klaiber [25] or the Jive system by Kleber [26]. Both systems are not yet mature enough to be compared with the CHILI environment. They lack basic image analysis functions, cooperative teleconferences, strong security features, and the full spectrum of vendor and system independent communications. The performance drawback of Java is another aspect which has to be taken into account; but independence from the operating system and the hardware are fascinating features.

### Conclusion and Outlook

CHILI is not just a research project where the system has been used for some test cases. It is integrated in the clinical routine in many different locations and institutions. The accounting numbers and the feedback of the users are proving that the CHILI architecture provides a powerful and flexible environment for PACS and teleradiology. PlugIn modules will also extend the multimodality workstation for teleradiology by providing advanced image analysis and therapy planning functionality.

In the future, development will continue on the Java implementation of the complete CHILI architecture to offer the most flexible and hardware/software independent environment for PACS and teleradiology. The feedback and suggestions of the users has influenced the system architecture by a great extent. Our own experience is that the differences between PACS and teleradiology components will vanish. This observation is supported by a recent market investigation by Frost&Sullivan [27]. The "tele" aspect will become a natural feature of radiological workstations. Radiologists will profit from better performance; and as a consequence, the patients will receive better treatment.
